# Epithelioid hemangioendothelioma of the forearm with radius involvement. Case report

**DOI:** 10.1186/1746-1596-6-120

**Published:** 2011-12-06

**Authors:** Claudia D Gherman, Daniela Fodor

**Affiliations:** 1 Surgical Clinic No. 2, "Iuliu Hatieganu" University of Medicine and Pharmacy, No. 4-6 Clinicilor Street, 400006 Cluj-Napoca, Romania; 2 Medical Clinic No. 2, "Iuliu Hatieganu" University of Medicine and Pharmacy, No. 4-6 Clinicilor Street, 400006 Cluj-Napoca, Romania

**Keywords:** vascular tumor, intermediate-malignant tumor, secondary bone involvement

## Abstract

Epithelioid hemangioendothelioma (EHE) is a rare, well-differentiated endothelial tumor with intermediate malignancy which develops more frequently from the peripheral veins, generally in the lower limb. Bone EHE comprises less than 1% of the bone neoplasms. We present the case of a young man, 24-year-old, with EHE of the forearm with secondary involvement of the distal radius. The location and the extension of the tumor allowed a wide excision, without the reconstruction of radius, followed by adjuvant radiotherapy, with a subsequent favorable evolution. Based on the clinical, radiographic, and pathological features of the EHE review, we concluded that it is difficult to adopt a standardized therapeutic approach due to the extremely low incidence of the bone involvement in EHE and the variable tendencies towards malignancy of this tumor. To our knowledge this is the third case of EHE with the involvement of the radius.

## Background

Epithelioid hemangioendothelioma (EHE) is a rare, well-differentiated endothelial tumor with a wide spectrum of behavior. EHE, also known as: low degree anaplastic angiosarcoma, cellular hemangioma, histiocytoid hemangioma or hemangioendothelioma, was considered a term meant to describe tumors that range between hemangiomas and angiosarcomas.

It represents 1% of all vascular neoplasms and it is locally aggressive [1].

Generally speaking, EHE as an extremely rare, intermediate-malignant vascular tumor, that develops from the peripheral veins of the lower limb, especially from the iliac and femoral veins [2]. The epithelioid hemangioendothelioma, an even less frequent vascular tumor, malignant by nature, is a tumor found in skull, spine, femur, tibia, and leg. The tumor is more frequent in men in the second- and the third-decade of life [1]. The surgical strategy differs depending on the primary localization of the tumor: excision, large resection with bone reconstruction, or amputation.

## Case presentation

### Clinical summary

**Figure 1:**
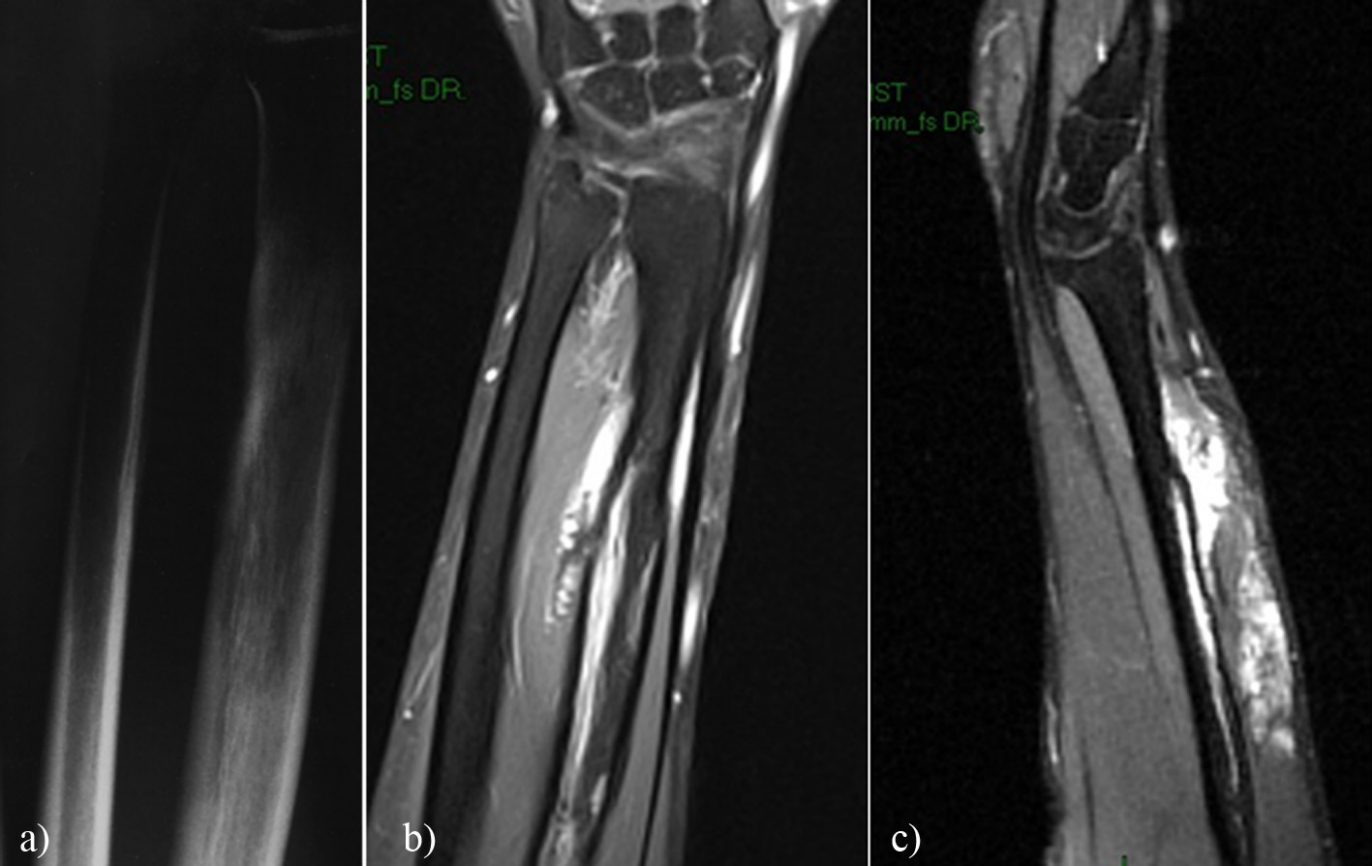
**X-ray and MRI forearm examination**. **1.a**. **AP radiograph of the distal forearm**. Lamellar periostosis of the radius bone associated with diffuse bone structure change and an area of reduced bone density (a) are seen. **1.b, c**. **MRI forearm examination**. Sagital FS T1 shows inhomogeneous tumor with irregular contours with diffuse post contrast enhancement and osseous invasion (b). MRI coronal section T1TSEfs shows post contrast better highlighting of the osseous invasion (c).

A 24-year-old, right-handed male, machinist, with no pathological personal or family history, was admitted to evaluate a right forearm mass that has slowly increased in the past 4 years. The patient described vague forearm pain, weakness and an oval mass (of approximately 7/4 cm) of hard-elastic consistency that was found on palpation of the extensor part of the forearm. No numbness or functional impairment of the hand were present and the neurovascular examination revealed a normal distal extremity. No history of trauma or other significant systemic diseases was found. Routine laboratory tests were in between normal range. The anteroposterior (AP) radiograph of the distal forearm showed the presence of lamellar periostosis of the radius bone associated with diffuse changes in the bone structure and an aria of reduced bone density in the distal radius (Figure 1a). The ultrasound performed described a highly vascularizated mass situated over the radius, poorly delimitated from the surrounding tissues. Magnetic resonance imaging (MRI) was obtained to delineate bone and soft tissue involvement (see details in Figure 1b and 1c).

**Figure 2:**
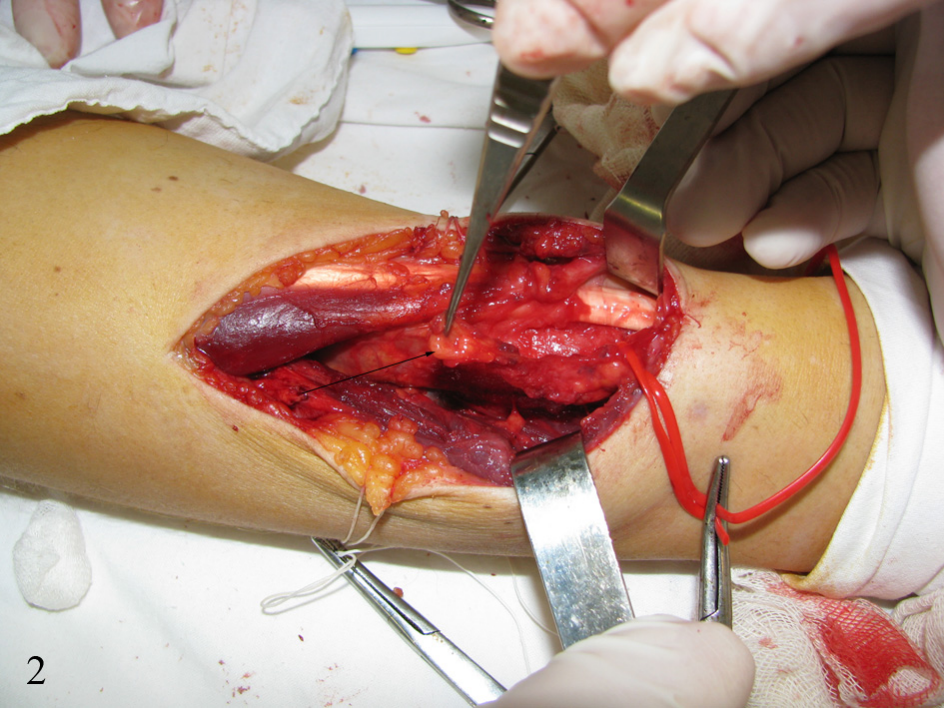
**Intraoperative detail**. EHE isolation from the forearm extensor compartment muscles, tumor invasion into adjacent radius being confirmed (arrow).

The patient was scheduled for elective surgical resection. Intrasurgically we found an oval mass that was delimitated by the extensor digitorum muscle, and the extensor brevis of the thumb muscle, and the tendinous portion of the extensor carpi radialis brevis muscle. (Figure 2). The tumor was not dissociable from the radius, the direct continuity with the bone substance being observed. This observation was interpreted as being a continuous tumor growing. Tumor invasion into the adjacent radius being confirmed, the lower third of the radius was scraped out.

### Pathological findings

The resected mass measured 6.8 cm × 3.5 cm and was surrounded by healthy tissue with grossly negative margins.

The submitted material was fixed in 10% formalin, paraffin-embedded and cut at 5 μm. Slides were stained with hematoxylin and eosin (H&E stain), Masson trichrome, with monoclonal antibody against CD31 (clone M0823, Dako Corporation), as well as the standard avidin-biotin complex immunostaining method for Ki67.

**Figure 3:**
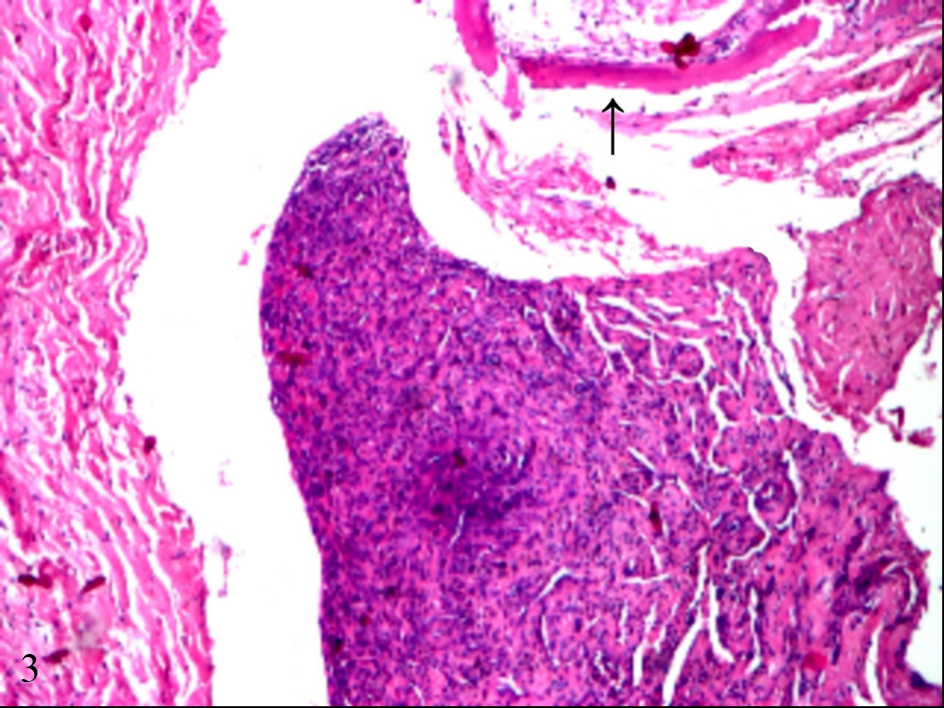
**Tumoral nodule in the vicinity of bone (arrow)**. H&E stain, original magnification × 10.

**Figure 4:**
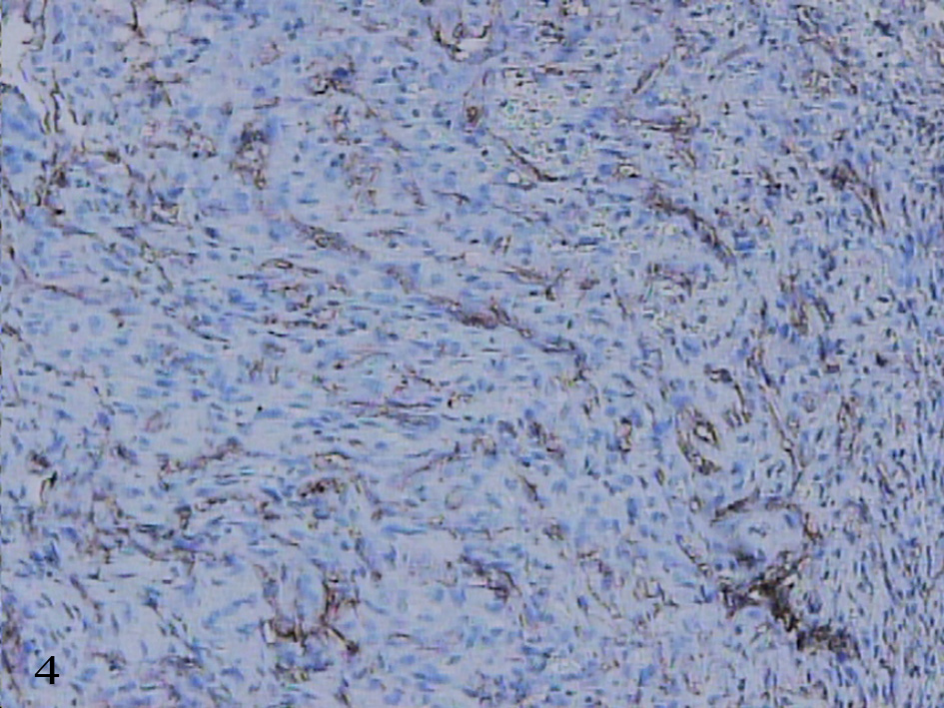
**Flattened cells, positive for anti-CD31 monoclonal antibody**. The vascular spaces are delineating within the tumoral nodule. Immunostaining, original magnification × 25.

**Figure 5:**
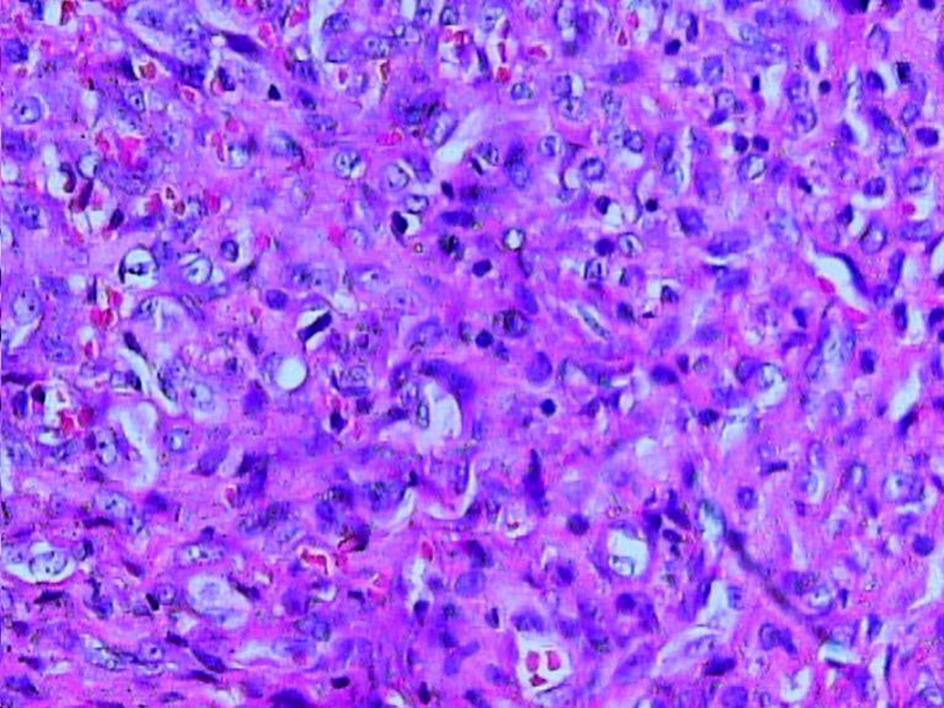
**Detail of a tumoral area cells with clear intracytoplasmic vacuoles**. Red blood cells are seen within the small vascular spaces. H&E stain, original magnification × 50.

Microscopic examination revealed the proliferation of venous channels with dilated lumina, partially filled with thrombi. Papillary endothelial proliferation was focally present. On this background, a particular finding was the presence of a solid intraluminal nodule, formed by spindled cells that infiltrated the venous wall and extended into the surrounding fibrous and adipose tissue, focally reaching the bone surface (Figure 3). The tumoral nodule showed many small angulated lumina, delineated by flattened cells, positive for CD31 (Figure 4). On higher magnification, the tumoral nodule was formed by spindled and epithelioid cells with mild nuclear atypia and scant mitotic activity. Prominent clear vacuoles were seen within the cytoplasm of some epithelioid cells (Figure 5).

The patient was referred to the Oncology Centre for local adjuvant radiotherapy having a total dose of 60 Gray/23 fractions/43 days. The tolerance of the treatment was good.

No regional or distant metastasis was found. No local recurrence has been observed at the 6, 12 and 24 month follow-up.

## Discussions

Besides EHE, the goup of hemangioendotheliomas includes the kaposiform Dabska tumor (papillary intralymphatic angioendothelioma), spindle-cell, retiform (hobnail hemangioendothelioma), and composite [3-5]

EHE is a rare neoplasm of the vascular endothelium, which was described for the first time in 1982 by Weiss and Enzinger as a subgroup of the hemangioendotheliomas having an important endothelial or histiocytic component [6]. Weiss and Enzinger, publishing their 46-patient series, reported for EHE a recurrence rate of 13% and a development rate of 31% for local-regional lymph metastases in the course of a 48-month follow-up period. On the same series the mortality rate was 13% [3,7]. The subsequent published series were smaller, so that the EHE mortality rate ranged between 0 and 20% in the case of monitorization periods between 3.5 and 4.4 years [3,8,9]. These initial studies were followed by case reports regarding other localizations, such as hepatic, pulmonary [10], and bone. In 55% cases EHE is multicentric and in 40% cases several bones are involved [3,8].

Clinically, EHE is accompanied by pain and swelling. On radiological examination, EHE is an expansive, osteolytic and poorly demarcated lesion. EHE occurs in male\ adults during the second or third decade of life, 60-80% involving the femur, tibia, fibula, or metatarsals [9,11]. Multiple lesions may be present at the level of one single bone. Also, in the bone, EHE is found in the metaphysis or epiphysis. Pathological fractures may also occur [1].

The preoperative evaluation of the EHE must be as complete as possible. The clinical examination should be completed with extensive imagistic investigations. Ultrasonography emphasizes its vascularization and allows a differential diagnosis vis-à-vis an arteriovenous malformation or even an aneurysm. Taking into consideration the rarity of the malignant character of bone EHE, especially at the upper limb level, the soft tissue sarcoma is the closest diagnosis that would come into discussion. The radiology examination and the MRI are necessary in order to determine the degree of the neoformation, the relations with the surrounding tissues, the possible existence of a cleavage plan, and the morphological data about the lesion [2].

The definite EHE diagnosis is established after a careful histopathological extensive differential diagnosis with Langerhans cell histiocytosis, angiosarcoma, infection, myeloma, metastasis, and lymphoma, intranodal palisaded myofibroblastoma, and other tumors of the hand and forearm region [12-14]. Since malignancy is only rarely presumed preoperatively, full body CT scans and/or positron emission tomography survey to detect the presence of local-regional or distant metastasis are conducted just as seldom [2].

Because the cytology prior to the surgical treatment cannot always confirm the malignant character, the treatment of choice is the complete local excision of the tumor with or without regional lymphadenectomy [2]. The amputation of the extremity of the limb is necessary only in the cases where the tumor has a high degree of infiltration. This attitude, namely the amputation above the elbow, was adopted in the case reported by Lai et al. [15] of a Kasabach-Merritt syndrome, characterized by a malignant hemangioendothelioma to which thrombocytopenia, hemolytic anemia, and consumption coagulopathy have been associated. The case reported by Duncan et al. [3] as an expandable tumor of the distal third of the pathologically fractured radius and which, after resection, required a radius reconstruction, is a conclusive example of bone EHE, the second after Tsuneyoshi [8], among which, out of a series of 14 reported patients with EHE, only in the case of 1 patient was the radius involved.

The particularity of our case consists in the surgical solution that did not require radius reconstruction or even upper limb amputation, similar to the other two cases. The subsequent favorable evolution in time of our case is an argument in favor of the correctness of the selected complex diagnostic and therapeutical attitude, the latter of which combined surgery and radiotherapy. To the best of our knowledge, we are describing the third case of EHE with distal radius involvement.

A standardized therapeutic approach is difficult to adopt due to the extremely low incidence of bone involvement or primitive bone EHE (under 1% of the total number of vascular tumors) and also because of the variable tendencies thereof towards malignancy [16].

The role of radiotherapy remains controversial, because of the same reason very few series of reported patients. In the series of Kleer et al. [8] 4 out of 10 patients who underwent radiotherapy for EHE had a favorable evolution, and Rosenthal et al. [17] mentioned the efficaciousness of radiotherapy in a case of a patient with multifocal bone EHE. The role of adjuvant chemotherapy, on the other hand, in the treatment of EHE, has not been established so far [8,18].

In summary, bone EHE, the destructive local level tumor with a variable evolution, is remarkable because of the diagnostic difficulties, both from a clinical and a histopathological point of view, resulting in a high frequency of initial misdiagnosis. Its behavior cannot be predicted based only on the histological characteristics, the most important criteria in the appreciation of an unfavorable prognosis seems to be the visceral implication [8]. Under these circumstances, and taking into consideration the particular situation of the reported case, in our opinion, EHE should be investigated via skeletal screening. When the osteolytic lesions involve more than 50% of the cortex there is a serious risk for pathological fractures [11]. Our case is in accordance with the previous reports as far as the treatment conduct is concerned, consisting in complete large excision followed by radiotherapy and subsequent monitoring.

## Consent

Written informed consent was obtained from the patient for publication of this Case Report and any accompanying images. A copy of the written consent is available for review by the Editor-in-Chief of this journal.

## Abbreviations

CT: Computer Tomography; EHE: Epithelioid hemangioendothelioma; MRI: Magnetic resonance imaging.

## Declaration of competing interests

The authors declare that they have no competing interests.

## Authors' contributions

CG conceived and designed the case report and drafted the manuscript. DF supplied clinical data and contributed to the revisions and editing of the manuscript. All authors have read and approved the final manuscript.
